# Heat Resistance of *Listeria monocytogenes* in Dairy Matrices Involved in Mozzarella di Bufala Campana PDO Cheese

**DOI:** 10.3389/fmicb.2020.581934

**Published:** 2021-01-06

**Authors:** Annalisa Ricci, Marcello Alinovi, Francesco Martelli, Valentina Bernini, Alessandro Garofalo, Giampiero Perna, Erasmo Neviani, Germano Mucchetti

**Affiliations:** ^1^Department of Food and Drug, University of Parma, Parma, Italy; ^2^Research and Development, Consorzio Tutela Mozzarella di Bufala Campana DOP, Caserta, Italy

**Keywords:** food safety, *L. monocytogenes*, thermal resistance, dairy matrices, stretching, *D*-value, *z*-value

## Abstract

The presence of *Listeria monocytogenes* in Mozzarella di Bufala Campana Protected Designation of Origin cheeses may depend on curd stretching conditions and post contaminations before packaging. To avoid cross-contamination, thermal treatment of water, brines and covering liquid may become necessary. The present study aimed to improve knowledge about *L. monocytogenes* thermal resistance focusing on the influence of some cheese making operations, namely curd stretching and heat treatment of fluids in contact with cheese after molding, in order to improve the safety of the cheese, optimize efficacy and sustainability of the processes. Moreover, the role that cheese curd stretching plays in *L. monocytogenes* inactivation was discussed. The 12 tested strains showed a very heterogeneous heat resistance that ranged from 7 to less than 1 Log_10_ Cfu/mL reduction after 8 min at 60°C. *D*-values (decimal reduction times) and *z*-values (thermal resistance constant) calculated for the most heat resistant strain among 60 and 70°C were highly affected by the matrix and, in particular, heat resistance noticeably increased in drained cheese curd. As cheese curd stretching is not an isothermal process, to simulate the overall lethal effect of an industrial process a secondary model was built. The lethal effect of the process was estimated around 4 Log_10_ reductions. The data provided may be useful for fresh pasta filata cheese producers in determining appropriate processing durations and temperatures for producing safe cheeses.

## Introduction

Fresh pasta filata cheeses have been usually considered among the microbiologically safe cheeses because of the supplementary effect of the stretching of the acid cheese curd with hot or boiling water. Nonetheless, Mozzarella di Bufala Campana (MBC) Protected Designation of Origin (PDO) cheese is characterized by pH (>5.1) and water activity (a_*w*_ > 0.975) values that do not allow to automatically consider it as food not able to support *Listeria monocytogenes* growth, according to Commission Regulation (EC) No. 2073/2005 (2020). From 1998 to 2017 out of more than 370 cases of cheese microbial contamination, only three cases of Mozzarella withdrawn from the market because of the presence of *L. monocytogenes* have been reported by European Rapid Alert System for Food and Feed (RASFF Portal, 2020). Suddenly, in 2018 and 2019 three recalls of MBC occurred in France (RASFF 2018/1198 and 2018/2459) and in Canada (Canadian Food Inspection Agency (CFIA), 2019), because of the presence of *L. monocytogenes*. This unusual contamination has urgently raised the need to investigate on its possible reasons, since the risk profile of MBC may be considered lower than the more widely consumed cow milk Mozzarella, because of the higher acidity of the cheese curd (pH 4.8 vs pH 5.1) and the higher temperature of the stretched curd at the end of the process (about 68–72°C vs 58–63°C; Mucchetti and Neviani, 2006). The stretching process of buffalo cheese curd is associated to significant but extremely variable decimal reductions of *L. monocytogenes* count, ranging from 1 to 8, as resulted by some studies performed with laboratory scale cheese making trials (Villani et al., 1996; Raimundo et al., 2013; Serraino et al., 2013; Murru et al., 2018). The survival probability depends on the cheese curd contamination, the effective conditions of stretching applied during the experiments, often not fully described, and the heat resistance of biotypes. Up to present, at the best of our knowledge, no *L. monocytogenes* contamination has been reported in MBC cheese curds. Its presence at the moment of stretching is believed to be occasional and lower than the counts measured during challenge tests, where artificial curd contamination ranged from 10^3^ to 10^7^ CFU/g. In these challenge tests, *L. monocytogens* was added to milk, and during cheese curd maturing a decrease (Murru et al., 2018) or even a small but significant growth (Serraino et al., 2013) have been observed. The degree of *L. monocytogenes* survival after stretching may be associated to the stretching conditions, as the overall amount of thermal and mechanical energy applied, that determine the temperature and time of permanence of the cheese curd at lethal temperature, is inversely related to the degree of cheese curd maturity and demineralization. Indeed, to obtain a cheese with the standard and typical MBC structure, the cheesemaker lowers the stretching temperature when the cheese curd has a too low pH and, as a consequence, an excessive extent of casein demineralization (Mucchetti and Neviani, 2006; Mucchetti et al., 2016).

Moreover, further aspects should be considered to explain the variable effects of curd stretching on *L. monocytogenes* survival. First, heterogeneity (genetic or phenotypic diversity) within a population, can promote adaptation and survival when the population experiences sudden environmental changes (Ryall et al., 2012; Davis and Isberg, 2016). These phenomena may explain the ability of a fraction of a bacterial population to withstand stresses that kill the majority of the population itself (Booth, 2002), and could be associated both to *Listeria* spp. biodiversity (Bernini et al., 2013) and to the presence of isogenic variants, able to influence its heat tolerance, e.g., because of a different ability to repair cell membrane after sub lethal heat injury (Somolinos et al., 2010). Heterogeneity can further contribute to explain the limits of the widely used primary model of Bigelow (Bigelow, 1921), when deviations from linear relationship between time of heat treatment and survival are observed (Cebrian et al., 2017). The heat resistance range of *L. monocytogenes* biotypes is quite variable, as reported by Doyle et al. (2001) and Aryani et al. (2015b), with *D*-values at 60°C ranging from few seconds to more than 4 min, and decreases at low pH, high a_*w*_ and low NaCl content (Jørgensen et al., 1995; Mazzotta, 2001; Bucur et al., 2018). Heat resistance of *L. monocytogenes* is also influenced by the growth phase, with the cells in exponential phase being more heat sensitive than those in stationary phase (Lou and Yousef, 1996; Jørgensen et al., 1999; Aryani et al., 2015b), and by stress adaptation phenomena, e.g., acid and or osmotic stress (Jørgensen et al., 1995; De Jesús and Whiting, 2003). Moreover, it may be interesting to note that some *D*- and *z*-values measured in some complex food matrices (meat, eggs) were significantly higher than those determined in culture media as Tryptone Soy Broth (TSB; Quintavalla and Campanini, 1991; Doyle et al., 2001), confirming the role of the interaction of microorganisms with the matrix in determining its stress tolerance.

Furthermore, the presence of *L. monocytogenes* in MBC can also be the result of post-contamination during the processing steps after stretching and before packaging. After stretching, MBC cheese is hardened and cooled by dipping into flowing tap water, salted by immersion in a brine and finally packed by dipping into a covering liquid, composed by salt and organic acids (e.g., lactic acid; Mucchetti and Neviani, 2006). Potentially, the contamination of these fluids can be transferred to the cheese, but at present time no data demonstrated the presence of *L. monocytogenes* in tap water and/or brines and/or covering liquids used for MBC cheese making. However, even if the presence of *L. monocytogenes* in tap water is not largely documented (Lyautey et al., 2007), its ability to survive (Budzińska et al., 2012), and to create biofilm (Gião and Keevil, 2014) has been determined. Otherwise, the presence of *L. monocytogenes* in brines of different cheeses has been detected using analytical tools as quantitative polymerase chain reaction (PCR) (Alessandria et al., 2010; Barancelli et al., 2014), and its ability to survive was inversely related to the acidity and NaCl content of the brine itself (Schirmer et al., 2014). The presence of this pathogen on cheese surface and/or on equipment may result in water and brine contamination, causing the beginning of a non-controlled cross-contamination. Also, the more frequent presence of *Listeria* spp. on non-food contact surfaces (floor, drains, walls, and platforms) may become a potential route of cheese contamination (Barancelli et al., 2014). To avoid cheese cross-contamination, the treatment of water, brines and covering liquid may become necessary. Beside heat processes, filtration, and/or UV-C treatment (Gayan et al., 2015) can be effective in reducing the count of *L. monocytogenes*. However, heat treatments can lead to more reproducible results.

Knowledge of decimal reduction time (*D*-value) and thermal resistance constant (*z*-value) in each out of these matrices can allow to build primary and secondary models able to foresee the lethal effect of heat treatments, contributing to improve the efficacy and the sustainability of the processes, avoiding to waste energy and/or time, mainly when batch treatments are applied as occur in many small sized MBC dairies. The *D*-value is the time required to obtain a ten-fold reduction of a microbial population at a constant lethal temperature and it varies according to the microorganism and to the medium where heat is applied. The *z*-value is the temperature variation required for the *D*-value to change by a factor of ten. Using these parameters, it is possible to foresee the effect of a thermal treatment, accepting that the death rate follows a first order reaction kinetics. Despite the death rate of many microorganisms does not always or completely follow this order of kinetics, suggesting that other models (e.g., Weibull frequency distribution models or non-log-linear Geeraerd’s model) could be applied (Peleg and Cole, 1998; Mafart et al., 2002; Geeraerd et al., 2005; Cebrian et al., 2017), the linear model (Bigelow, 1921) continues to offer a relevant contribution to predict the effect of a heat treatment. However, in the presence of a non-isothermal temperature profile, as in the case of curd stretching operation, it is necessary to develop a secondary model able to simulate the overall lethal effect of the treatment.

The aims of this study were (i) the selection of the most heat resistant *L. monocytogenes* among a pool of dairy origin strains, (ii) the estimation of *D* and *z* values of this strain using as matrices MBC cheese curd and the fluids coming into contact with MBC cheese after cheese molding, such as hardening water, brine and fresh covering liquid; (iii) the proposal of a secondary model able to estimate the cumulative effect of the stretching process on *L. monocytogenes* survival. Following this approach, we aimed to improve the knowledge about the influence of the matrix on heat resistance and to better understand the role that cheese curd stretching plays in *L. monocytogenes* inactivation.

## Materials and Methods

### Culture Preparation

Twelve *L. monocytogenes* strains were overall considered in this study. Among these, ten strains (Lm1, Lm2, Lm3, Lm4, Lm5, Lm8, Lm15, Lm16, Lm21, and Lm28) isolated from Gorgonzola cheese (Bernini et al., 2013) and belonging to the collection of Food and Drug Department of the University of Parma, and two reference strains (Lmg 21263 corresponding to Atcc 13932, isolated from a clinical patient affected by meningitis, and Lmg 13305 corresponding to Dsm 15675, isolated from soft cheese) purchased from Bccm (Belgian Co-ordinated Collections of Microorganisms) of Ghent University, Belgium were considered. The stock cultures were kept frozen at −80°C in Tsb (Vwr, Milano, Italy) added with 12.5% glycerol (v/v). They were recovered in TSB (VWR) enriched with 0.6% yeast extract (Oxoid, Basingstoke, United Kingdom) and incubated at 37°C overnight. The procedure was replicated three times. The counts after overnight growth were determined in Agar Listeria according to Ottaviani and Agosti (ALOA; VWR, Milano, Italy) incubated at 37°C for 48 h. Before being used for heat resistance experiments, microbial cells were separated by centrifugation (Centrifuge 5810 R, Eppendorf) at 12,857 *g* for 5 min at 25°C, washed twice in Ringer solution (Oxoid, Milan, Italy) and finally resuspended in Ringer solution to reach the proper cell concentration.

### Thermal Resistance Screening of *L. monocytogenes* Strains *in vitro* Conditions

The selection of the strain with the highest heat resistance was carried out as follows. Small volumes of Tsb (Vwr; 2.970 mL) were pre-heated in conical plastic tubes (50 mL capacity) in a water bath (Type M900-Ti Basic, Instruments s.r.l, Italy) until they reached the temperature of 60°C. Then 0.03 mL of each strain was inoculated in different pre-heated Tsb aliquots in order to obtain a final concentration ranged from 6 to 7 Log_10_ Cfu/mL. The inoculated solutions were rapidly mixed and kept for 8 min at 60°C. In order to obtain a rapid temperature decrease and stop the heat treatment the suspensions were chilled by immersion into a water bath with melting ice to fulfill the condition of iso-temperature, monitoring the temperature with type-K thermocouples. The initial and the residual microbial concentration were checked on ALOA (Vwr) at 37°C for 48 h. The detection limit was 1 Log_10_ Cfu/mL and the tests were performed in duplicate.

### Determination of *L. monocytogenes* Heat Resistance in TSB, Hardening Water, Brine, Covering Liquid, and Cheese Curd

The strain that showed the highest heat resistance was selected and further tested to estimate *D* and *z* values in the range from 60 to 70°C in TSB and in different matrices of MBC cheese production chain: hardening water, brine, covering liquid, and cheese curd. Matrices were provided by Consorzio Tutela MBC DOP, with these main characteristics:

(i) hardening water represents a sample of pasteurized tap water that was used to chill MBC in a continuous equipment without recycle of the water;
(ii) brine is a freshly prepared light brine (NaCl concentration 4 *g*/100 *g*, titratable acidity 6°Soxhlet-Henkel Sh/50 mL) used to salt MBC according to the rules of MBC production standard;
(iii) covering liquid is a pasteurized solution of 0.05 *g*/100 mL of lactic acid (titratable acidity 6°Sh/50 mL; pH 2.9);
(iv) cheese curd is mature MBC cheese curd (about 45% of moisture content, a_*w*_ 0.99) sampled before the stretching step and immediately frozen at −18°C.

A small volume (0.985 mL) of each fluid matrix was warmed at the selected temperature immerging conical plastic tubes (50 mL capacity) into a water bath (Type M900-Ti Basic, Instruments s.r.l, Italy) to reach the target equilibrium temperature. Then 0.015 mL of *L*. monocytogenes washed cell suspension was added; the inoculated fluid matrix was then rapidly mixed and kept for the scheduled time at the set temperature (that ranged from 60 to 70°C). The suspensions (1 mL) were chilled by dilution into 9 mL of Ringer solution (Oxoid) at 4°C. In this way, as checked by temperature monitoring with type-K thermocouples, the conditions of iso-temperature were fulfilled. The initial and residual counts were made on Aloa (Vwr) at 37°C for 48 h. The detection limit was 1 Log_10_ Cfu/mL.

The test in MBC cheese curd was performed introducing a step of preparation of the matrix, as its warming determines a whey syneresis. So, 10 *g* of cheese curd were poured into a conical plastic tube (50 mL capacity) and kept into a water bath at the set temperature (62 to 70°C) up to 30 min for completing the whey separation. Total solids and a_*w*_ of the drained cheese curd were measured according to Idf standard 4A (1982) and by AquaLab Water Activity Meter Series 3TE with internal temperature control (Decagon Devices, Inc., Pullman, WA, United States), respectively. Then 1.97 *g* of drained cheese curd was poured into a new conical plastic tube and, when equilibrated at the target temperature of treatment, 0.03 mL of *L. monocytogenes* washed cell suspension was inoculated directly into the hot mass and kept for the scheduled times. After treatment, instantaneous chilling to non-lethal temperature was obtained by the addition of 18 mL of Ringer solution (Oxoid) at 4°C. All the trials were performed in duplicate.

### Estimation of *D*- and *z*-Values for the Primary Model

The *D*-value, that is the absolute value of the inverse slope of the linear regression line between the Log_10_ of surviving cells number and time (s), was calculated considering at least three out of four or more measures performed at different times for each experiment and falling into the linear portion of the regression line. The absolute value of the inverse slope of the linear regression line between the average Log_10_ *D*-values and temperature was the *z*-value.

### Simulation of the Effect of Cheese Curd Stretching on *L. monocytogenes* Survival

In order to benchmark the results of *L. monocytogenes* thermal inactivation obtained in cheese curd, an exemplificative Mbc curd stretching process was considered according with temperature data obtained from observations made in Mbc dairies (Table 3). The process was divided into four key steps: melting caused by curd mixing with hot water, kneading of melted curd, cheese molding, and cooling (Table 3). To estimate the effect of cheese curd stretching step on *L. monocytogenes* survival, a secondary model was built applying the following procedure:

(1) $$D_{T_{X}}=D_{T_{\text{REF}}}\,10^{-\frac{T_{X}-T_{\text{REF}}}{z}}$$

(i) Estimation of *D*-values at all the lethal temperatures (Tx) involved in the process on the basis of Eq. 1, using a primary linear model, with T_*REF*_ = 70,0°C;
(ii) calculation of the estimated time of permanence of the coldest spot of the cheese curd at lethal temperature (e.g., from 60 to 70°C) both during the stretching operation and the initial step of cooling;
(iii) the time of permanence at each temperature was divided by the corresponding *D*-values to obtain the Log_10_ count reduction at each temperature;
(iv) finally, the sum of the lethal effects at each temperature represents the overall lethal effect given by the process.

## Results

### Selection of the Most Heat Resistant *L. monocytogenes* Strain

In general, the heat resistance of the 12 strains was heterogeneous. Among all the tested strains, Lm15 was the most heat resistant showing a decrease in cell concentration of less than 1 Log_10_ CFU/mL after 8 min of heating at 60°C in TSB. *L. monocytogenes* LMG 21264, isolated from a patient affected by meningitis, was the most heat sensitive strain; *L. monocytogenes* Lm3, Lm8, Lm21, Lm28, and LMG 13305 were the most sensitive among the strains isolated from cheese (Table 1).

**TABLE 1:** Log_10_ reduction of *L. monocytogenes* tested strains after 8 min at 60°C in *in vitro* conditions.

**Strain**	**Log_10_ CFU/mL**			
	**Trial A**	**Trial B**	**Mean**	**SD**
Lm1	1.86	1.56	1.71	0.21
Lm2	1.12	1.83	1.48	0.50
Lm3	5.28	5.31	5.29	0.03
Lm4	3.50	4.38	3.94	0.62
Lm5	3.21	2.76	2.98	0.32
Lm8	4.98	5.34	5.16	0.25
Lm15	0.52	0.72	0.62	0.14
Lm16	2.92	2.61	2.76	0.22
Lm21	4.55	6.20	5.37	1.17
Lm28	6.72	6.37	6.55	0.25
LMG 21264	7.08	7.04	7.06	0.03
LMG 13305	4.90	5.70	5.30	0.56

### Estimation of *D*- and *z*-Values of *L. monocytogenes* Lm15 Strain in Dairy Matrices

*D*-values of Lm15 estimated at temperatures ranging from 60 to 70°C in fluid media and between 62 and 70°C in drained cheese curd have been strongly affected by the matrix (Table 2). Considering the fluid matrices, the high presence of organic matter in TSB (31.5 g/L) appeared to protect the cells from heat damage at all three temperatures considered. The acidic pH (2.9) of covering liquid that was due to the presence of lactic acid contributed to negatively affect the heat resistance of Lm15; on the contrary, this strain showed a higher thermal resistance in the hardening water and in the brine. Heat resistance of Lm15 increased noticeably at all the tested temperatures when measured in the drained cheese curd, characterized by a total solid content of 63.4% and a_*w*_ value of 0.989, because of whey separation that was caused by the preliminary equilibration step to heating temperature. Even the *z*-values were affected by the different matrices, with values much higher for the dairy matrices (about 10°C) compared to the synthetic medium TSB (6.4°C).

**TABLE 2:** *D*-values and *z*-values of *L. monocytogenes* Lm15 in different matrices.

**Matrix**	**pH**	**Temperature (°C)**	***D*-value (s)**	***z*-value (°C)**
TSB	7.2	60.10.3	284.417.3	6.4
		67.30.1	19.32.3	
		70.20.6	8.40.3	
Hardening water	7.2	60.00.2	90.75.0	8.2
		67.40.2	6.90.1	
		70.20.2	6.10.4	
Brine	3.2	60.00.1	84.44.9	10.1
		67.50.2	16.40.4	
		70.40.1	7.70.1	
Covering liquid	2.9	60.10.2	22.10.7	9.8
		67.20.1	4.30.1	
		70.20.2	2.10.2	
Drained cheese curd	5.1	62.30.4	423.526.6	11.1
		66.40.1	188.919.4	
		70.00.3	85.47.2	

### Simulation of the Effect of Cheese Curd Stretching on *L. monocytogenes* Survival

As stretching is not a fully isothermal operation, its lethal effect on Lm15 should be foreseen based on the temperature profile of the curd. On the basis of the hypothesized curve temperature vs cumulative time (Table 3), four steps of the process contributing to the overall lethal effect can be identified. The cumulative lethal effect of the first three steps, where mixing and kneading contribute to a roughly homogeneous heating rate, can be estimated around 4 Log_10_ reductions (3.92 Log_10_ in the example) of Lm15 strain (Table 3). In the center of the MBC cheese ball, being the slowest point to be chilled, the lethal effect can increase to more than 6 Log_10_ count reduction. Conversely, in the external part of the cheese (which temperature decrease is not estimated in Table 3) because of the very fast cooling to sublethal temperature the count reduction does not overcome the one obtained during stretching (∼4 Log_10_ count reduction). To foresee the overall lethal effect of the thermal treatment, the zone where the effect is lower has to be considered. Furthermore, the outer zone of the cheese is the most subjected to post-contamination by contact with hardening water, brine, and covering liquid.

**TABLE 3:** Estimation of the overall lethal effect of cheese curd stretching on Lm15 (D_70^°c_ = 85.36 s; *z* = 11.1°C).

	**Estimated Lm15 D_*T*_ (s)**	**Temperature (°C)**	**Estimated time of curd residence at “constant” temperature (s)**	**Cumulative time of stretching operation (s)**	**Log_10_ count reduction**	**Log_10_ count reduction cumulative**
Curd mixing with hot water	681	60	3	3	0.00	0.00
	553	61	4	7	0.01	0.01
	449	62	6	13	0.01	0.02
	365	63	8	21	0.02	0.05
	297	64	10	31	0.03	0.08
	241	65	15	46	0.06	0.14
	196	66	20	66	0.10	0.24
	159	67	25	91	0.16	0.40
	129	68	30	121	0.23	0.63
	105	69	45	166	0.43	1.06
Kneading of melted curd	105	69	200	366	1.90	2.97
Cheese molding	105	69	100	466	0.95	3.92
Cheese cooling and hardening	129	68	80	546	0.62	4.54
	159	67	80	626	0.50	5.04
	196	66	65	691	0.33	5.37
	241	65	60	751	0.25	5.62
	297	64	50	801	0.17	5.79
	365	63	40	841	0.11	5.90
	449	62	30	871	0.07	5.97
	553	61	30	901	0.05	6.02
	681	60	30	931	0.04	6.06

## Discussion

A large variability of heat resistance among the *L. monocytogenes* strains tested was observed in this study and it has to be taken into account in future studies on *L. monocytogenes* survival during heat processes of food products. Our results in TSB medium agree with previously reported data (Doyle et al., 2001; Aryani et al., 2015b). A similar high heat resistance was found by Quintavalla and Campanini (1991) for a strain isolated from meat and tested in the same matrix. Curiously, the most heat resistant strain among the 20 tested by Aryani et al. (2015b), with the majority of them of food origin, was isolated from milk. Otherwise, some strains showed a very low heat resistance; the most heat sensitive strain was a reference strain (LMG 21264, isolated from human meningitis), whose heat resistance was about 11 times lower than that of Lm15 and similar to Lm28 that was about 10 times less heat resistant than Lm15. De Jesús and Whiting (2003) found a narrower distribution from 1.98 to 5.31 min of the D_60^°c_ values among 21 *L. monocytogenes* strains isolated from humans and animals, while the range for the same acid-adapted strains was between 0.8 and 4.59 min.

The influence of the matrix on the heat resistance of the selected strain used in the present study was clear and it was probably linked to a combination of protective and inhibiting factors. The high amount of total solids of the drained cheese curd (63.4%) and of the synthetic TSB medium (31.5 g/L) may be related to the high *D*-values measured at all the tested temperatures. No literature data are available about the heat resistance of *L. monocytogenes* in MBC cheese curds, while D_60^°c_ values in TSB (4.74 min corresponding to 284.4 s in Table 2) resulted similar to those reported by Quintavalla and Campanini (1991) and Aryani et al. (2015b). A higher thermotolerance of *L. monocytogenes* in half cream, double cream or butter compared to TSB was reported by Casadei et al. (1998), but no relationship was found between the *D*-values and the fat amount of the product. The presence of undissociated lactic acid in the covering liquid may be responsible for the lowest *D*-values reported in the present study. As the fresh covering liquid contained 0.5 *g* of lactic acid/100 mL at pH 2.9, according to the Henderson-Hasselbalch equation.

The amount of undissociated acid is near to 90% corresponding to about 50 mmol/L, largely above the concentration of 6.35 mM indicated by Aryani et al. (2015a) and Wemmenhove et al. (2018) as inhibitory of *L. monocytogenes* growth. Considering the values obtained in hardening water and brine, the relatively low NaCl concentration (4 *g*/100 *g*) of the latter fluid did not markedly affect Lm15 heat resistance, probably because the protective effect of salt was counterbalanced by the low pH value of the brine. The direct relation between salt concentration and *L. monocytogenes* thermotolerance was observed by several authors (Jørgensen et al., 1995; Juneja et al., 2013; Li et al., 2017).

The calculation of *z*-values between 60 and 70°C in the different matrices gave results generally higher (from 8.2 to 11.1°C) than most of the data present in literature, with the exception of *z*-value related to TSB medium (6.4°C). However, similar or higher *z*-values were found for *L. monocytogenes* strains tested in some foods such as egg albumen or yolk, meat or flour (Palumbo et al., 1996; Michalski et al., 2000; Taylor et al., 2018). A higher *z*-value means that the strain is more tolerant to the changes in temperatures and this behavior should be taken into account in the design of heat processes.

As the knowledge of *D*- and *z*-values is pivotal to successfully design food processes, the accuracy of the simulation of the lethal effect due to stretching and other heating operations depends both on the accuracy of the *D*- and *z*-value estimation, both on the accuracy of the determination of the heat treatment. While temperature changes during fluid heating by flowing through continuous heat exchangers can be predicted by computational fluid dynamics (Rinaldi et al., 2018) or direct measurements, the measurement of the change of temperature of a cheese curd during continuous cooking-stretching operation in industrial conditions, by means of the typical diving arms equipment used in the geographical area, is difficult to be monitored or estimated, and for this reason is lacking in literature. Cheese curd stretching is considered as an asymmetrical heat treatment (Villani et al., 1996; Murru et al., 2018) and just few known examples (Serraino et al., 2013) of description of the temperature evolution are referred to lab scale experiments or to industrial trials performed with batch operations (Mucchetti et al., 1997). The process involves solid/fluid mixing operations, rheological changes due to curd melting, and a flow in a channel with not constant section, responsible for a variable speed and residence time in the different sections of the equipment (tween screw conveyor, diving arms, and molding). The process can be divided in four steps: (i) a first step of fast rise of temperature of the curd mass, which homogeneity depends on the efficacy of cheese curd mixing with hot water into the horizontal counter rotating twin screw conveyor that transports the mixture to the kneading section; (ii) a second step where the melted cheese curd is kneaded by the movement of diving arms and temperature is quite constant and homogenous within the mass; (iii) a third step where the stretched cheese mass enters the molding equipment and it is pulled by a second screw conveyor toward the molding drum; (iv) a final step during which the molded cheese balls fall from the molding drum into flowing tap water where they start to harden and to decrease their temperature; the temperature decrease in the center of the cheese, that is slower than in its external part, is governed by the conductive heat transfer from the center to the surface of the cheese and it is dependent of the cheese ball size. The minimum overall sterilizing effect on *L. monocytogenes* is the result of the cumulative effect of non-isothermal (the steps of curd mixing with hot water and cheese chilling by dipping in tap water) and quasi-isothermal (the steps of curd kneading and cheese molding, performed without water addition) contributions. The accuracy of lethal effect prediction depends on the correct measurement of the temperature profile of the coldest spot in the non-isothermal steps of the process. This discussion shows the complexity of the phenomenon of heat exchange during the curd stretching operation and the difficulties to foresee the rate of inactivation of microorganisms during cheese curd stretching and cheese hardening. At the same time, beside the strain biodiversity (Murru et al., 2018), the potential process variability can contribute to explain the heterogeneous results obtained by challenge tests, where the inactivation of *L. monocytogenes* ranged from 1 to more than 8 decimal reductions (Villani et al., 1996; Kim et al., 1998; Raimundo et al., 2013; Serraino et al., 2013; Murru et al., 2018). The technological importance of bacterial heterogeneity is also connected to the ability of a small fraction of any population to survive exposure to stresses that kill the majority of the population. The knowledge of heat inactivation kinetics parameters for all the other fluids involved in chilling, salting and storing of MBC cheese is critical to more effectively and efficiently manage the treatment of these fluids to prevent MBC cheese surface post contamination.

## Conclusion

The data supplied in this *in vitro* study may prove to be useful for MBC and other Mozzarella cheese producers in determining appropriate durations and temperatures for producing fresh pasta filata cheeses avoiding the presence of *L.* monocytogenes. To do so, a further model should be studied to foresee the amount of hot water required to heat the MBC cheese curd mass, considering both the cases of batch or in continuous stretching processes. Finally, the knowledge of the kinetic parameters of heat inactivation together with a deeper knowledge of the temperature vs time evolution of the cheese mass can be a tool to foresee the lethal effect of the process and to better manage the process itself also With the aim to improve the microbial safety of the cheese.

## Data Availability Statement

The raw data supporting the conclusions of this article will be made available by the authors, without undue reservation.

## Author Contributions

AR: investigation and methodology. MA: methodology and data analysis. FM: investigation and methodology. VB: project administration, supervision, and writing -review and editing. AG: resources. GP: resources. EN: supervision, writing -review and editing. GM: conceptualization, supervision, and writing – original draft. All authors contributed to the article and approved the submitted version.

## Conflict of Interest

The authors declare that the research was conducted in the absence of any commercial or financial relationships that could be construed as a potential conflict of interest.
